# Genome-Wide Profiling of Prognostic Alternative Splicing Pattern in Pancreatic Cancer

**DOI:** 10.3389/fonc.2019.00773

**Published:** 2019-08-27

**Authors:** Min Yu, Weifeng Hong, Shiye Ruan, Renguo Guan, Lei Tu, Bowen Huang, Baohua Hou, Zhixiang Jian, Liheng Ma, Haosheng Jin

**Affiliations:** ^1^Department of General Surgery, Guangdong Provincial People's Hospital, Guangdong Academy of Medical Sciences, Guangzhou, China; ^2^Department of Medical Imaging, The First Affiliated Hospital of Guangdong Pharmaceutical University, Guangzhou, China; ^3^The Second School of Clinical Medicine, Southern Medical University, Guangzhou, China

**Keywords:** alternative splicing, TCGA, pancreatic cancer, prognosis, driver gene

## Abstract

Alternative splicing (AS) has a critical role in tumor progression and prognosis. Our study aimed to investigate pancreatic cancer-specific AS events using RNA-seq data, gaining systematic insights into potential prognostic predictors. We downloaded 10,623 genes with 45,313 pancreatic cancer-specific AS events from the Cancer Genome Atlas (TCGA) and SpliceSeq database. Cox univariate analyses of overall survival suggested there was a remarkable association between 6,711 AS events and overall survival in pancreatic cancer patients (*P* < 0.05). The area under the curves (AUC) of the receiver operator characteristic curves (ROC) of risk score was 0.89 for final prognostic predictor. Results indicated that AS events of DAZAP1, RBM4, ESRP1, QKI, and SF1 were significantly associated with overall survival. The results of FunRich showed that transcription factors KLF7, GABPA, and SP1 were the most highly related to survival-associated AS genes. Furthermore, using DriverDBv2, we identified 13 driver genes associated with survival-associated AS events, including TP53 and CDC27. Thus, we concluded that the aberrant AS patterns in pancreatic cancer patients might serve as prognostic predictors.

## Introduction

During the pre-mRNA splicing, introns are removed, and the exons are left to form the final mRNA products. In this process, exons which are left vary, and thus, one single gene may generate multiple mRNA isoforms by alternative splicing (AS). More than 95% of human genes undergo AS, and most of them vary in levels across different cells and tissues (1). Variations in AS may result in a spectrum of consequences from completely functional inactivation, to subtle or difficult-to-detect effects, or possibly to altering the location, stability or translation of a transcript, including oncogenes and tumor-suppressor genes. Alternative splicing has not only critical roles in normal development but also is indispensable in multiple pathological processes, including cancers (2–4). Previous studies have provided evidence that aberrant splicing patterns are closely related to tumor progression and prognosis (2). For example, alternative splicing in pre-mRNA of Epidermal Growth Factor Receptor (EGFR) produces several isoforms, some of which are constitutively active, leading to enhanced tumorigenicity, migration, and invasion (5, 6). EGFR, Insulin Receptor (INSR), and Vascular Endothelial Growth Factor Receptor (VEGFR), whose alternative splicing features variated, result in promoting tumor progression or reduced response to therapy (7). Recent evidence found that several tumor suppressor genes undergo aberrant AS in cancer, which leads to either complete or partial loss of function, such as TP53 (8). Therefore, alternative splicing events might be ideal biomarkers for cancer diagnosis and prognosis and even be served as a potential target which might help scientists to discover new drugs.

The conventional molecular method for quantification of AS is a reverse transcription polymerase chain reaction (RT-PCR). There are several other techniques, including expressed sequence tags (ESTs) and splicing-sensitive microarrays, which were invented to identify the connections between genotypes and AS patterns in patients. However, these technologies have low throughput, high noise, or restrained to known splicing events. Powered by high-throughput RNA-seq, the amount of human transcriptome data has grown tremendously over the past decade, and large-scale studies in aberrant AS events at a more fine-grained level are now available. Recent advances in RNA-Seq and related bioinformatics methods allow researchers and clinicians to discover cancer-related AS and further investigate the molecular mechanism.

Pancreatic cancer is still known as one of the most malignant solid tumors whose 5-year survival rate has remained under 8% over the past 30 years. The disease is typically found at a late stage when the resection is impossible. Moreover, a response rate of only one-quarter or less can be expected, and resistance of current chemotherapy, such as gemcitabine, occurred in most of the pancreatic cancer patients. At present, the molecular mechanism of pancreatic cancer development and progression is still unclear. Researches have been undertaken to elucidate the mechanisms of this malignancy, including AS in specific gene transcription (9–11). However, few studies have tried to investigate the prognostic value of AS in pancreatic cancer. Therefore, the present study identified pancreatic cancer-specific AS events by analysis of RNA-seq data downloaded from The Cancer Genome Atlas (TCGA) program, gaining more information about their functions in cancer biology in detail.

## Materials and Methods

### Alternative Splicing Events From TCGA RNA-Seq Data

**Figure 1:**
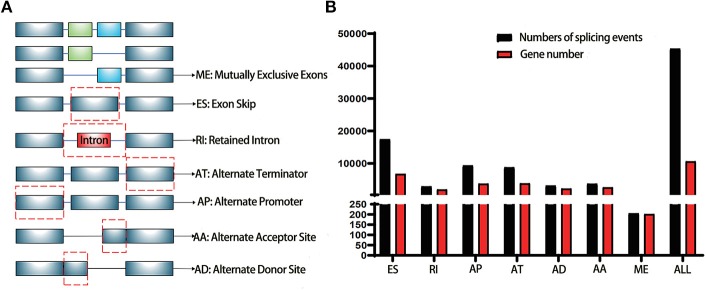
Illustrations for alternative splicing during seven types in this study. **(A)** Schematic example of AS events, ME, Mutually exclusive exons; ES, Exon skip; RI, Retained intron; AT, Alternate terminator; AP, Alternate promoter; AA, Alternate acceptor site; AD, Alternate Donor site; **(B)** A number of AS events and involved genes from TCGA PAAD cohort were depicted according to the AS types. The black bar represents the preliminarily detected AS events. The red bar represents the related genes.

TCGA (https://tcga-data.nci.nih.gov/tcga/) is a landmark cancer genomics program with a large amount of detailed information across various cancers in public database (12). The RNA-Seq data of pancreatic cancer cohorts (PAAD) was downloaded for further analysis. SpliceSeq (http://bioinformatics.mdanderson.org/TCGASpliceSeq) is a Java application which explores the mRNA alternative splicing patterns of TCGA data. The SpliceSeq tool was used to investigate the mRNA splicing pattern of PAAD samples from the TCGA database. SpliceSeq aligned reads to available transcripts of genes in the Ensembl database and built a unified splice graph. Then, the PAAD sample reads are aligned to the splice graph, and the feature of splicing for each transcript will be summarized. The Percent Spliced in (PSI) value is a parameter to assess the chance of each splicing event. There are several subtypes of splice events: Exon Skip (ES), Alternate Promoter (AP), Mutually Exclusive Exons (ME), Alternate Terminator (AT), Retained Intron (RI), Alternate Donor site (AD), and Alternate Acceptor site (AA). The detailed information of each subtype of splicing event in PAAD was shown in Figure 1A.

### Survival Analysis

Clinical information of the PAAD cohort with 178 patients was available in the TCGA database (12). Summary characteristics of these patients were shown in Supplementary Table 1. In order to build the model and further analysis, we used mean values to replace the null value in the dataset of the splicing events. For each AS event, the patients were divided into two groups according to the median value; then the Univariate Cox analyses were performed to identify survival- associated splicing AS events in pancreatic cancer (*P* < 0.05). The Multivariate Cox regression was performed to determine the prognostic value of splicing events (*P* < 0.05). Then, the most significant top 20 genes in each model were chosen for the forest plots. Above analyses were performed using R/Bioconductor (version 3.5.2) and SPSS (version 25.0).

### Construction of the Model of Risk Scores

Predictive models were built with prognostic events from identical AS subtype, respectively, whereas the final model was constructed with the whole splicing events from PAAD. In order to evaluate accuracy of model of risk scores, we drew the K-M curve, and the cut-off value is *P* < 0.01. Receiver-operator characteristic (ROC) curves were drawn, and the values of the area under the curves (AUC) were used to compare the predictive power of each model. All analyses were performed using R/Bioconductor (version 3.5.2) and Graphdpad Prism 8.0.

### UpSet Plot and Gene Network Construction

Intersections between different types of AS were investigated by UpSet R (13). UpSet R is a novel R package which provides intersecting sets using matrix design, along with visualizations of several common sets, element, and attribute related tasks. Gene Ontology (GO) and Kyoto Encyclopedia of Genes and Genomes (KEGG) pathway were performed and were significant when the *P*-value was <0.05 in KEGG and 0.0001 in GO analysis. GO Enrichment plot were used to depicted gene interaction network, function annotation, and pathway enrichment of survival- associated AS genes. Therein, using Cytoscape (version 3.7.1), significant genes with the smallest *P*-value in univariate analysis were selected for the drawing of the PPI network.

### Splicing Correlation Network Construction

The expression of splicing factor genes in mRNA splicing pathway was investigated by analysis of the level 3 mRNA-seq data in TCGA. Pearson correlation test was used to analyze the correlation between the mRNA expression of splicing factor gene and the PSI value of survival- associated alternative splicing events. Cytoscape (version 3.7.1) was used to construct the interaction network of the significant genes with the smallest *P*-value.

### Analysis of Splicing-Factor, Transcription Factors, and Driver Gene

The association between survival- associated AS events and splicing factors was further investigated. Firstly, the log-rank test was used to identify survival- associated splicing factors. The list of 71 known splicing factors was extracted from the SpliceAid 2 (https://bioinformatics.mdanderson.org) database, which was released in February 2013 (14). The expression profiles of splicing factors were downloaded from the TCGA database and further converted into transcripts per million (TPM). Pearson correlation test was applied to assess the association between survival-associated AS and survival- associated splicing factors. FunRich (Functional Enrichment analysis tool for transcription factors) from ExoCarta (http://www.exocarta.org/), DriverDBv2 (A database for human cancer driver gene research) and David (http://david.abcc.ncifcrf.gov/) databases were used to perform the analysis. To find the correlation between gene mutation status and AS events, *t-*test was performed. Pearson correlation test was also performed to investigate the association between mRNA expression of driver genes and AS events. R software (version 3.5.2) was applied for bioinformatics analysis, and *P* < 0.05 was considered significant (Two-sided tests).

## Results

### Number of mRNA Splicing Events in PADD Cohort From TCGA

**Figure 2:**
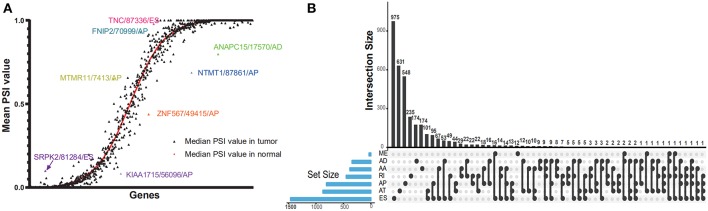
Dot plot and UpSet plots in PAAD. **(A)** Correlation between tumor PSI and normal PSI in splicing factors were depicted in the dot plot. The smooth red curve was drawn according to median PSI value in normal; the black triangles represented the median PSI value of genes in the tumor. **(B)** The UpSet intersection diagram shows seven subtypes of splicing associated AS events in PAAD. One gene might have more than one subtype of survival-associated AS event.

The PSI value of all the splicing events was calculated by SpliceSeq. To identify each AS event precisely, each AS event was named by gene name followed by the unique as_ID and AS types. For example, for the name S100A13/7733/AP, S100A13 is the gene name, 7733 is the as_ID in the dataset, and the AP is the AS subtype. As depicted in Figure 1B, a total of 10,623 genes with 45,313 AS events were detected in 178 pancreatic samples, including 17,402 ESs in 6,750 genes, 2,873 RIs in 1,922 genes, 9,325 APs in 3,724 genes, 8,733 ATs in 3,816 genes, 3,118 ADs in 2,210 genes, 3,657 AAs in 2,594 genes, and 205 MEs in 202 genes. Overall results showed that one gene might have an average of 4.2 AS events. Among those genes, 8,833 genes had more than one type of AS events. Gene collagen type 1 alpha 1 (COL1A1) had the maximum number of AS events (*n* = 484), followed by mitochondrial ribosomal protein L55 (MRPL55) (*n* = 74) and interleukin 32 (IL32) (*n* = 68). Among those splicing subtypes, ES was the main subtype of AS events, while ME was relatively rare in the tumor. Besides, only a small proportion of AS events (1,622 out of 45,313) were novel splice. The PADD cohort of TCGA also included four normal samples; the PSI median values of different genes were also summarized and further analyzed. Several genes splicing events, including KIAA1715/56096/AP, ZNF567/49415/AP, NTMT1/87861/AP, ANAPC15/17570/AD, SRPK2/81284/ES, MTMR11/7413/AP, FNIP2/70999/AP, and TNC/87336/ES, differed significantly between tumor and normal samples (Figure 2A). When compared to normal samples, cancer samples had reduced alternative splicing diversity (41,629 AS events in normal vs. 40,959 in cancers).

### Survival-Associated AS Events in PAAD Cohort

**Figure 3:**
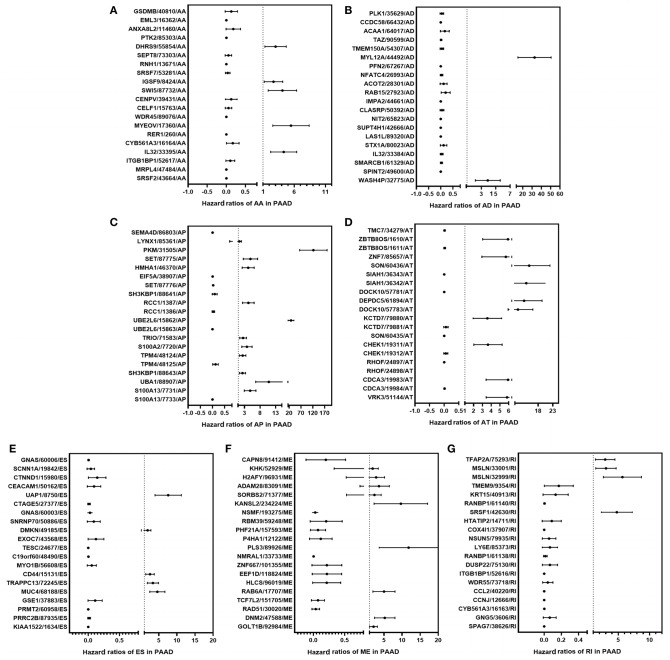
Forest plots show the top 20 survival-associated AS events of the seven AS subtypes, respectively. The circles represent HRs in the plots; Horizontal bars represent 95% CIs. Forest plots of HRs for survival associated AA subtypes **(A)**, AD subtypes **(B)**, AP subtypes **(C)**, AT subtypes **(D)**, ES subtypes **(E)**, ME subtypes **(F)**, RI subtypes **(G)** in PAAD.

**Figure 4:**
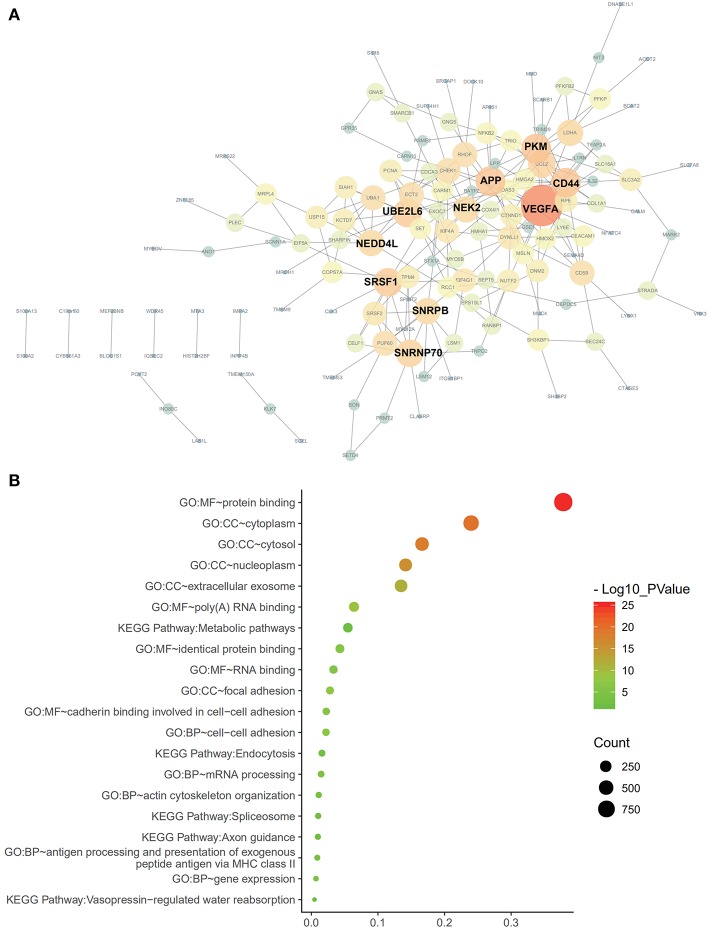
Protein-protein interaction analysis and gene enrichment in PAAD. **(A)** Survival-associated AS events interaction network created by Cytoscape. Genes are represented as nodes in the plot, and their interactions were denoted by lines. The size and color of the nodes represent Degree values and change pattern, respectively. The gene of lighter color and greater circle shows the higher Degree values in this network, whereas the darker color and the smaller circle show the smaller Degree values in this network. **(B)** Pathways identified by GO and KEGG analyses. Top 15 enrichment analysis of GO (include BP, CC, and MF, respectively) and top five pathways KEGG analyses of genes from OS-related alternative splicing events. GO, Gene Ontology; KEGG, Kyoto Encyclopedia of Genes and Genomes; CC, cellular component; Mf, molecular function; BP. Biological process.

Cox univariate analyses of overall survival were applied to explore survival- associated AS events in PAAD cohort. The results showed that 6,711 AS events strongly correlated with OS (*P* < 0.05), including 550 RIs from 449 genes, 421 AAs from 382 genes, 385 ADs from 342 genes, 1,499 APs from 809 genes, 1,649 ATs from 873 genes, 2,174 ESs from 1,463 genes, 33 MEs from 33 genes and 550 RIs from 449 genes. The UpSet plot was a novel method to display the intersecting sets, which may be more intuitive and superior to the Venn diagrams. As depicted in the plot, most of these genes had two or more AS subtypes associated with survival, but none of them possessed seven AS subtypes simultaneously (Figure 2B). The top 20 survival-associated AS events of the seven AS subtypes were presented in Figure 3. In top 300 genes from survival-associated AS events, some genes were top hub genes in the network, such as VEGFA, CD44, pyruvate kinase gene (PKM), amyloid beta precursor protein (APP), ubiquitin-conjugating enzyme E2 L6 (UBE2L6) (Figure 4A). In pancreatic cancer, KEGG pathway analysis showed that “Metabolic pathway,” “Endocytosis,” and “Axon guidance” were most significantly enriched by these genes. GO analysis revealed that “Protein binding,” “poly(A) RNA binding,” and “RNA binding” in molecular function, “cytoplasm,” “cytosol,” and “extracellular exosome” in cellular component, “cell-cell adhesion,” “mRNA processing,” and “actin cytoskeleton organization” in biological process were the most significantly enriched (Figure 4B).

### Prognostic Models for PADD Cohort

**Figure 5:**
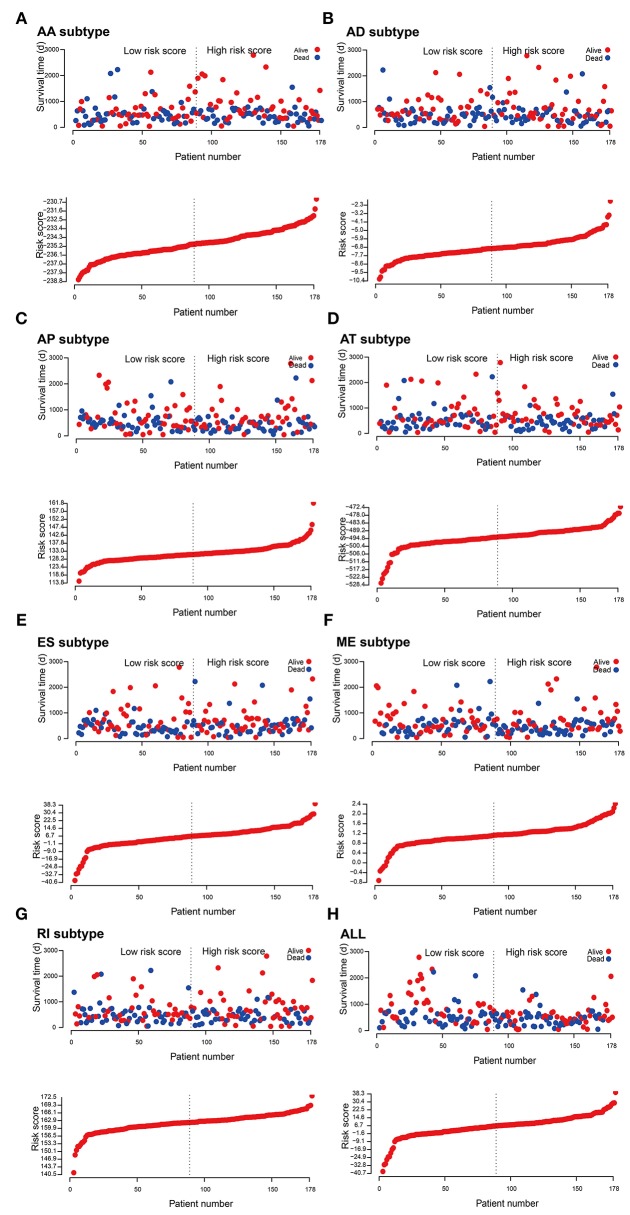
Construction and analysis of risk score based on the survival-associated splicing events using multiple Cox regression analysis. PAAD patients were divided into low- and high-risk groups based on the median value of risk score. The top of each assembly drawing represents survival status and survival time of PAAD patients distributed by risk score, the bottom part is the risk score curve of patients with PAAD. Risk scores were constructed using **(A)** AA subtypes, **(B)** AD subtypes, **(C)** AP subtypes, **(D)** AT subtypes, **(E)** ES subtypes, **(F)** ME subtypes, **(G)** RI subtypes, and **(H)** ALL subtypes of survival-associated splicing events.

**Figure 6:**
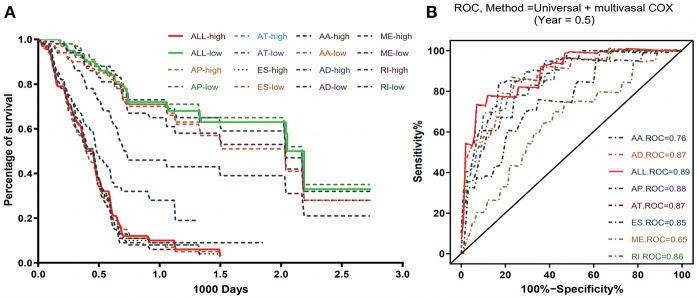
Kaplan-Meier and ROC curves of prognostic predictors in PAAD cohort. **(A)** Kaplan-Meier plot depicting the survival difference between the high and low-risk group in these prognostic models. **(B)** ROC analysis for all prognostic models. The different color lines of ROC curves represent different subtypes of AS events.

To evaluate the prognostic value of AS events in pancreatic cancer, the survival-associated AS events were selected to construct the prognostic risk score models in each subtype of AS events (Figure 5). As depicted in the results, all of the models showed significant value to predict the outcome of pancreatic cancer patients, including RI subtype (*P* < 0.0001), ES subtype (*P* < 0.0001), AP subtype (*P* < 0.0001), AT subtype (*P* < 0.0001), AA subtype (*P* < 0.0001), ME subtype (*P* < 0.0001), and AD subtype (*P* < 0.0001) (Figure 6A). The final prognostic model was built by a combination of prognostic AS events from different subtypes and showed significant prognostic value in distinguishing high-risk patients (*P* < 0.0001). Notably, the final prognostic model showed better performance than seven AS subtypes. The final prognostic predictor had the highest predicting efficiency analyzed by ROC (AUC = 0.89), followed by the AP model in subtypes (AUC = 0.88) (Figure 6B).

### Network of Survival-Associated Splicing Factor, Transcription Factors, and Driver Gene

**Figure 7:**
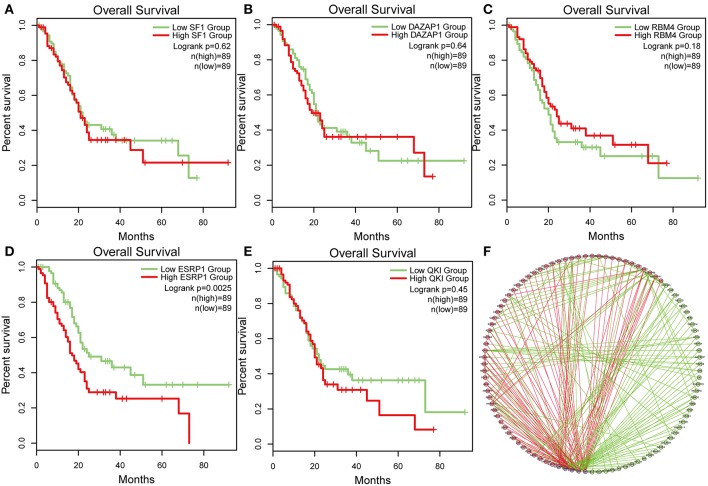
Survival-associated splicing factors and splicing correlation network in PAAD. **(A–E)** The prognostic value of mRNA expression of five splicing factors expression, whose AS events was significantly associated with overall survival in PAAD. **(F)** Splicing correlation network in patients with PAAD constructed by Cytoscape. These five splicing factors (purple dots) were positively (red lines) or negatively (green lines) associated with AS events, which predicted good (green dots) or poor (red dots) outcomes in patients with PAAD.

To identify survival-associated splicing factors, we performed a survival analysis about splicing factors based on PSI values. A total of 71 splicing factors from the SpliceAid2 database were chosen for survival analysis. Results showed that AS events of five splicing factors, including DAZ associated protein 1 (DAZAP1), RNA-binding motif 4 (RBM4), Epithelial Splicing Regulatory Proteins 1 (ESRP1), Quaking (QKI), and steroidogenic factor 1 (SF1), significantly associated with overall survival. The level 3 RNA sequence data were downloaded from TCGA, and the correlations of splicing factors expression and survival were analyzed. As depicted in Figures 7A–E, the expression of ESRP1 (*P* = 0.0025) significantly associated with survival, but DAZAP1 (*P* = 0.064), QKI (*P* = 0.45) and SF1 (*P* = 0.62) and RBM4 (*P* = 0.18) were not. The association between PSI values of top significant AS events and survival-related splicing factors was still unknown. Thus, String tool was used to investigate the association and gain systematic insights into their interaction. Only genes that are significantly related to each other were included in the network. In the correlation network, there was a significant association between the expression of five survival-associated splicing factors and 95 survival-associated AS events. Among 95 survival-associated AS events, 56 AS events (green dots) predicted good survival, whereas 39 AS (red dots) events strongly associated with poor survival in pancreatic cancer (Figure 7F). Correlation between these five splicing factors and representative AS events was shown in dot plots, suggesting the potential association between them (Supplemental Figure 1).

**Figure 8:**
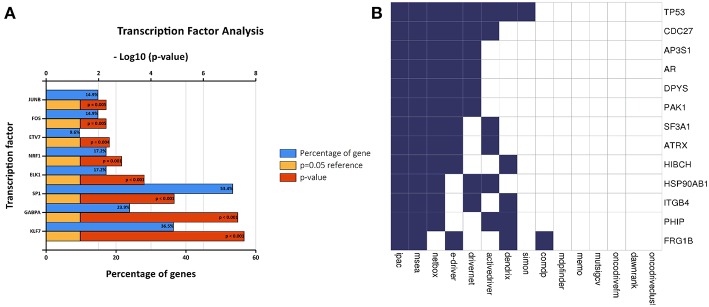
Correlation between transcription factors, driver mutation and splicing factors. **(A)** The histogram shows the results of transcription factor prediction from survival- associated AS events. The blue band represents the gene percentage, the yellow band represents the *P*-value standard (*P* = 0.05), and the red band represents the *P*-value. **(B)** A list of driver genes was generated by at least five bioinformatics tools using the DriverDB.

A transcription factor enrichment prediction performed among the survival-associated AS events using the FunRich software. Results identified several transcription factors, including Krüppel-like factor 7 (KLF7), GA binding protein transcription factor subunit alpha (GABPA), trans-acting transcription factor 1 (SP1), that might be the most significant transcription factors associated with survival-associated AS events. Transcription factor SP1 was the most highly related to 53.4% of all the survival- associated AS genes, followed by KLF7 (36.5%) and GABPA (23.9%) (Figure 8A).

**Figure 9:**
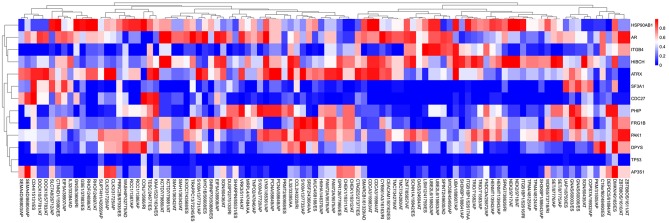
The correlation between PSI value of AS events and mutated status of driver genes was explored through *t*-test. Colors represented the *P*-value of *t*-test. Blue to red means *P*-value from low to high.

A list of driver genes was generated by at least five bioinformatics tools using the DriverDB, which is a database for the investigation of cancer driver gene and mutations. Results showed that 13 driver genes were identified, including tumor protein p53 (TP53), which were previously reported (15) (Figure 8B). In the mutation profile of driver genes, mutation of TP53, FSHD region gene 1 family member B (FRG1B), and cell division cycle 27 (CDC27) occurred in most of PAAD cohort from TCGA. As for the mutation class, truncating and missense were the two main types for driver genes, such as TP53, FRG1B, and CDC27 (Supplemental Figure 2). In addition, we investigated the correlations between mRNA expression of driver genes and the top 30 survival-associated AS events. Results indicated that mRNA expression of adaptor-related protein complex 3 subunit sigma 1 (AP3S1), integrin subunit beta 4 (ITGB4), and p21 (RAC1) activated kinase 1 (PAK1) was significantly associated with most of the top 30 survival-associated AS events (Supplemental Figure 3). Samples were divided into several groups according to numbers of driver gene mutations, and results indicated that numbers of AS events for each sample were not significantly associated with numbers of driver gene mutations (Supplemental Figure 4). Furthermore, we explored the correlation of AS events and mutation profiles by the *t*-test and found that mutation status of TP53, splicing factor 3a subunit 1 (SF3A1), and CDC27 significantly correlated with most of the Top-100 survival-associated AS events (Figure 9).

## Discussion

Alternative splicing enables a single gene to generate multiple mRNAs. Moreover, these mRNAs can be translated into various proteins with diverse functions and structures. Emerging data have demonstrated that aberrant AS patterns were identified in various cancers and engaged in multiple carcinogenic processes during cancer development and progression (16). The previous study demonstrated the AS events of tissue factors promoted neovascularization and monocyte recruitment via integrin ligation, thus contributing to activation of coagulation and tumor spread in pancreatic cancer (17). In pancreatic cancer, AS events of the PKM were differentially regulated and promoted the expression of the PKM2 isoform. Compared to PKM1, switching PKM2 AS events is beneficial to withstand gemcitabine and cisplatin-induced genotoxic stress, thus induced chemoresistance (18). Serine and arginine-rich splicing factor 1 (SRSF1) and heterogeneous nuclear ribonucleoprotein K (hnRNPK) were aberrantly upregulated in pancreatic cancer, leading to the increased expression of anti-apoptotic splice variants of Bcl-x and Mcl-1, significantly affected responses to chemotherapy (19). Previous data concerning the function of AS events in pancreatic cancer mainly focused on one or several genes, and there was no study which had explored the prognostic value of AS comprehensively. Given the importance of AS events in cancer, we investigated AS events and gained a comprehensive insight into the prognostic value of AS events in pancreatic cancer through the analysis of TCGA.

Among the genes with AS events, Gene COL1A1, which makes part of a large molecule called type I collagen, have the maximum number of AS events. Further analysis revealed some of COL1A1 AS events significantly correlated with survival. Our results were consistent with previous studies (20–22). Evidence showed that COL1A1 could activate β1-integrin and the activation, along with the epithelial-mesenchymal transition, contributed to the development of PAAD (23). The previous study has also demonstrated that once PAAD cells met COL1A1, Snail expression conducted by the increasing of TGF-β1 (Transforming Growth Factor- β1) signaling would begin, which in turn accelerate the progress of PAAD invasion by the upregulated MT1-MMP (membrane type 1-MMP) expression (24). Evidence also showed that hypoxia augmented the transcription and deposition of COL1A1 by TGF- β pathway, and COL1A1 was identified as a hypoxia marker in the non-small cell lung carcinoma (20). Abnormal COL1A1 lead to increasing radioresistance in cervical cancer and had its potential prognostic value in gastric cancer (21, 22). However, the implication of dysregulated splicing pattern of COL1A1 in cancer, including pancreatic cancer with abundance fibrosis, remains to be elucidated. When compared to normal samples, cancer samples had reduced alternative splicing diversity. A previous study reported that the splicing factor genes were upregulated in seven cancer types, including colorectal adenocarcinoma, breast cancer, and lung adenocarcinoma, while they were downregulated in four cancer types, including lymphoma and uterine cancer (2). In our study, we found that the total expression of the splicing factor genes in pancreatic cancer was downregulated. The results indicated that dysregulated expression of the splicing factor genes among cancer types was not in a fixed mode, which may partly result from tumor heterogeneity. Thus, systemic evaluation of the AS patterns in pancreatic cancer contributes to the understanding of the underlying mechanism of tumor development and progression.

Survival analysis was conducted, and interaction analysis between these survival-associated genes was performed. Results indicated that VEGFA closely related to other genes and served as a hub gene in the network. Among the VEGFA AS events, patients with VEGFA/76330/ES had better survival, implying that loss of Exon8 may weaken or abolish the interaction of VEGFA with other proteins and then inhibit the growth of the tumor. However, VEGFA/76336/ES significantly associated poor survival in pancreatic cancer, which is inconsistent with previous data (25). Of note, VEGFA/76336/ES, whose splice occurred with removal of exon7.1 and exon7.2 loss, lack the neuropilin binding site at exon7. In breast cancer, the VEGF-A/Neuropilin 1 pathway promoted cancer stemness by activating Wnt/β-Catenin axis, resulting in cancer stem cell phenotypes and chemoresistance (25). In acute myeloid leukemia, high expression of VEGFA was identified as an oncogenic factor, whose function may be reversed by SEMA3A competing for neuropilin (26). Theoretical speaking, removal of exon7, the binding site of neuropilin at VEGF sequence, abolish the interaction and inhibit tumor growth. However, VEGFA/76336/ES significantly associated unfavorable prognosis, which indicating its multifaceted roles in pancreatic cancer progression. It is hard to conclude that VEGFA/76336/ES promotes tumor growth due to a lack of experimental evidence. Nevertheless, our results indicated that neuropilin mediates cancer cell growth may rely on pathways independent of VEGFA. Additionally, blocking neuropilin may strengthen the role of anti-VEGF therapy in reducing the formation of new blood vessels. It is difficult to judge whether a gene is a cancer suppressor or a promoter since different AS events have varied, even opposite biological functions. Therefore, mRNA expression of a gene may be not adequate to determine the biological function, and the predominant AS events need to be taken into account.

Due to the characteristics of pancreatic cancer, including late diagnosis and poor outcome, several researchers had proposed some prognostic models based on mRNA, lncRNA, and microRNA (4, 27, 28). Nevertheless, seldom of these prognostic models come into widely used in clinical practices. Several studies published before finding that alternatively spliced variants contributed to cancer metastasis, cell cycle progression, and chemoresistance (18, 29, 30). As events have been previously identified as diagnostic, predictive, and prognostic biomarkers in pancreatic cancer (18, 31, 32). However, current knowledge about AS events was mostly derived from small samples studies or mainly focused on one single gene. Recently, a systemic analysis of AS events in pancreatic cancer was available due to high-throughput sequencing analysis and data from TCGA. Analysis of each subtype of splicing events was performed and found some of the AS events were of significant prognostic value in pancreatic cancer. Unlike other cancers, including colorectal cancer, lung cancer, the majority of AS events were closely associated with favorable prognosis in pancreatic cancer, especially in AD and RI subtypes. Prediction models were further built by each subtype, respectively or a combination of these seven subtypes. Among the models built by identical subtype, AP events demonstrated the highest efficiency in the prediction of survival outcome than other six subtypes. Moreover, the final prediction model built by a combination of seven subtypes showed better performance than other prediction models, with an AUC of ROC reaching 0.89 in distinguishing poor survival outcome. Our current work is the first to provide a comprehensive and systemic analysis of AS events and risk score models based on survival-associated AS events in pancreatic cancer.

The network of survival-associated splicing factors was evaluated and found AS events of DAZAP1, RBM4, ESRP1, QKI, and SF1 were significantly associated with overall survival, but the only mRNA expression of ESRP1 correlated with overall survival. Therefore, investigation into the AS events is important to judge the function of gene products. Epithelial-mesenchymal transition (EMT) is defined as a process that epithelial cells with tight junctions acquire a mesenchymal phenotype (33). This means that epithelial cells become easily mobile after this transition, that is, EMT can regulate metastasis (34). ESRP1 is a critical regulator in the epithelial splicing program through targeting several genes, such as fibroblast growth factor receptor 2 (FGFR2) and CD44 (also called H-CAM) (35, 36). As the levels of the mRNA of ESRP1 is down-regulated, the CD44 variant isoform is replaced by the CD44 standard isoform which promotes EMT, increasing invasiveness in gallbladder cancer (37). Evidence showed that the role of inflammation-inducible Snail in the driving malignant transformation of both normal and at-risk human bronchial epithelial cells required the silencing of RNA splice regulator ESRP1 (38). However, the evidence about the function of ESRP1 in pancreatic cancer still lacks and further studies are required. Current evidence has pointed out that splicing factors can precisely bind to a splice-regulatory sequence located at the gene, thus control the process of splicing (39). According to the difference in the sequence and structure, these splicing factors can be divided into two families, including Ser/Arg rich proteins (SR proteins) and the heterogeneous nuclear ribonucleoproteins (hnRNPs). By binding to sequence silencers or enhancers of splicing, these two families possess the opposite function in the mRNA splicing. However, the potential regulatory network of splicing factors during the splicing process remains unclear and clarifying the function of ESRP1 is critical in the interpretation of the molecular mechanism of pancreatic cancer. More attention should thus be paid to the study of AS events in pancreatic cancer.

The transcription process can impact AS events by a variety of mechanisms. Transcription factors can regulate the recruitment of splicing components, and modulate Pol II elongation rate, which regulates the kinetics of exposure of competing for splice sites (40). We evaluated the association between survival-associated AS events and transcription factors. Transcription factors KLF7, GABPA, and SP1, were the most highly related to survival-associated AS genes, which implied that one transcriptions factor might participate in splicing control of several genes. Krüppel-like factors (KLFs) was involved with many cellular activities, such as proliferation and metabolism (41–43). Moreover, a previous study reported that KLF7 transcriptionally activated argininosuccinate lyase, which resulted in polyamines production and the oncogenesis of glioma (44). KLF7 can also contribute to the migration and epithelial-mesenchymal transition of oral squamous cell carcinoma (45). However, the mechanism of how transcriptions factors engaged in the process of splicing is still unknown. It is reasonable that one single transcriptions factor may regulate several genes not only by direct binding to the promoter of targeted genes but also by indirect impact on splicing process.

Recent evidence showed that several genetic mutations, including K-Ras, TP53, SMAD family member 4 (SMAD4), and cyclin dependent kinase inhibitor 2A/P16 (CDKN2A/P16), drove the oncogenesis of pancreatic cancer (46). Except for these four driver genes, more and more genes are identified as the critical genes in the process of pancreatic cancer, including ret proto-oncogene (RET), AT-rich interaction domain 1A (ARID1A), and ATM (47). Driver genes have been identified as the building blocks in pancreatic cancer, and emerging data suggested that driver gene K-Ras involved in the process of splicing control, such as mucin 6 (MUC6), hepatocyte growth factor (HGF), VEGFR-2, and VEGFB (48). The abnormal expression of splicing factors of SR and hnRNP families results in dysfunction of targeting apoptotic genes, including p53 (19). However, rare studies had been conducted in the exploration of the association between driver genes and AS events. Potential driver genes were identified by the bioinformatic tool in the present study. Further analysis revealed that splicing events of each gene did not increase with accumulating gene mutations. Though the expression of TP53 and SF3A1 correlated with rare survival-associated AS events, mutation status of these two driver genes significantly correlated with many of the top 100 survival-associated AS events. SF3A1, which belong to candidate U2-dependent spliceosome genes family, was identified as driver genes by five prediction tools. Previous studies indicated that two SNPs (rs5994293 and rs9608886) of SF3A1, locating to the region of 22q12.2, were strongly correlated with pancreatic cancer (49). However, the mechanism of how driver genes, including SF3A1, lead to increasing AS events is still unclear. Our study findings enriched our knowledge about the mutation status of driver genes and regulation of splicing, gaining systemic insight into the molecular mechanism underlying PAAD.

Several limitations should be considered when interpreting the results. First, the included number of the PAAD samples was relatively small, and only four normal samples were available for PSI analysis. Second, the prognostic value of survival-associated AS events lack the external independent validation cohort. Third, the present study only investigated the data from high-throughput genomic sequence; experimental validation should be performed in the future.

In conclusion, our comprehensive investigation first focused on the aberrant AS patterns in pancreatic cancer and may contribute to the improvement of pancreatic cancer management and broaden to the novel field of prognosis and targeted molecular implications.

## Data Availability

The original data of the present study can be found at TCGA (https://tcga-data.nci.nih.gov/tcga/) and the SpliceAid 2 (https://bioinformatics.mdanderson.org).

## Author Contributions

MY contributed to conception and design, and acquisition, analysis, and interpretation of data. WH contributed to the acquisition of data of acquisition and data analysis. SR contributed to the acquisition of data, analysis, and interpretation of data. RG has been involved in drafting the manuscript and revising it critically. LT contributed significantly to drafting the manuscript. BHu contributed to acquisition of data. BHo contributed to revising the manuscript. ZJ contributed to interpretation of data. LM contributed to data interpretation. HJ conducted the study. All the authors participated in the discussion and editing of the manuscript.

### Conflict of Interest Statement

The authors declare that the research was conducted in the absence of any commercial or financial relationships that could be construed as a potential conflict of interest.

## Supplementary Material

Supplemental Figure 1 Correlation between these five survival-associated splicing factors and representative AS events was shown in dot plots.

Supplemental Figure 2 The mutation profile of 13 driver genes. The red band represents truncating, the purple band represents missense, and the green band represents inframe.

Supplemental Figure 3 The heatmap of the correlations between the mRNA expression of driver genes and PSI values of top 30 survival-associated AS events. Colors represented the correlation coefficient *r*.

Supplemental Figure 4 Samples from PAAD cohort were divided into several groups according to numbers of driver gene mutations from 0 to 12 in X-axis. No sample has thirteen gene mutations concurrently. The Y-axis represents the numbers of AS events of each sample.

Supplementary Table 1 Baseline characteristics according to TCGA Clinical data.
