# Supplementary figures and images for: Genome-Wide Profiling of Prognostic Alternative Splicing Pattern in Pancreatic Cancer
Source: Front Oncol. 2019 Aug 27;9:773. doi: 10.3389/fonc.2019.00773 (PMC6736558; doi:10.3389/fonc.2019.00773)

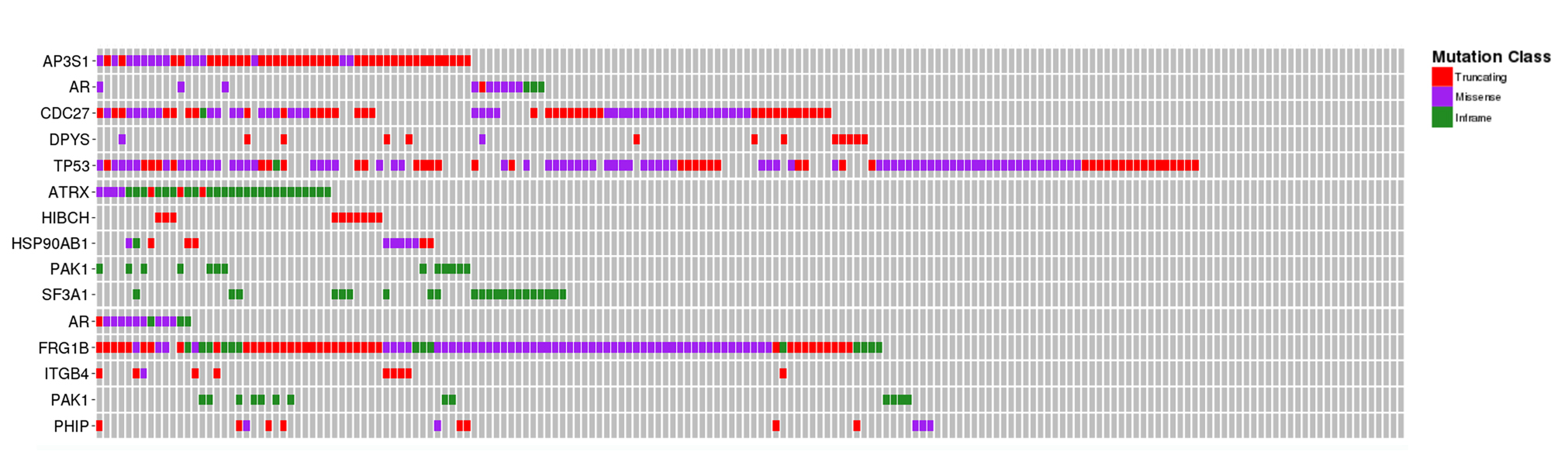

Supplement: Supplemental Figure 2 — The mutation profile of 13 driver genes. The red band represents truncating, the purple band represents missense, and the green band represents inframe. [file Image_2.TIF]

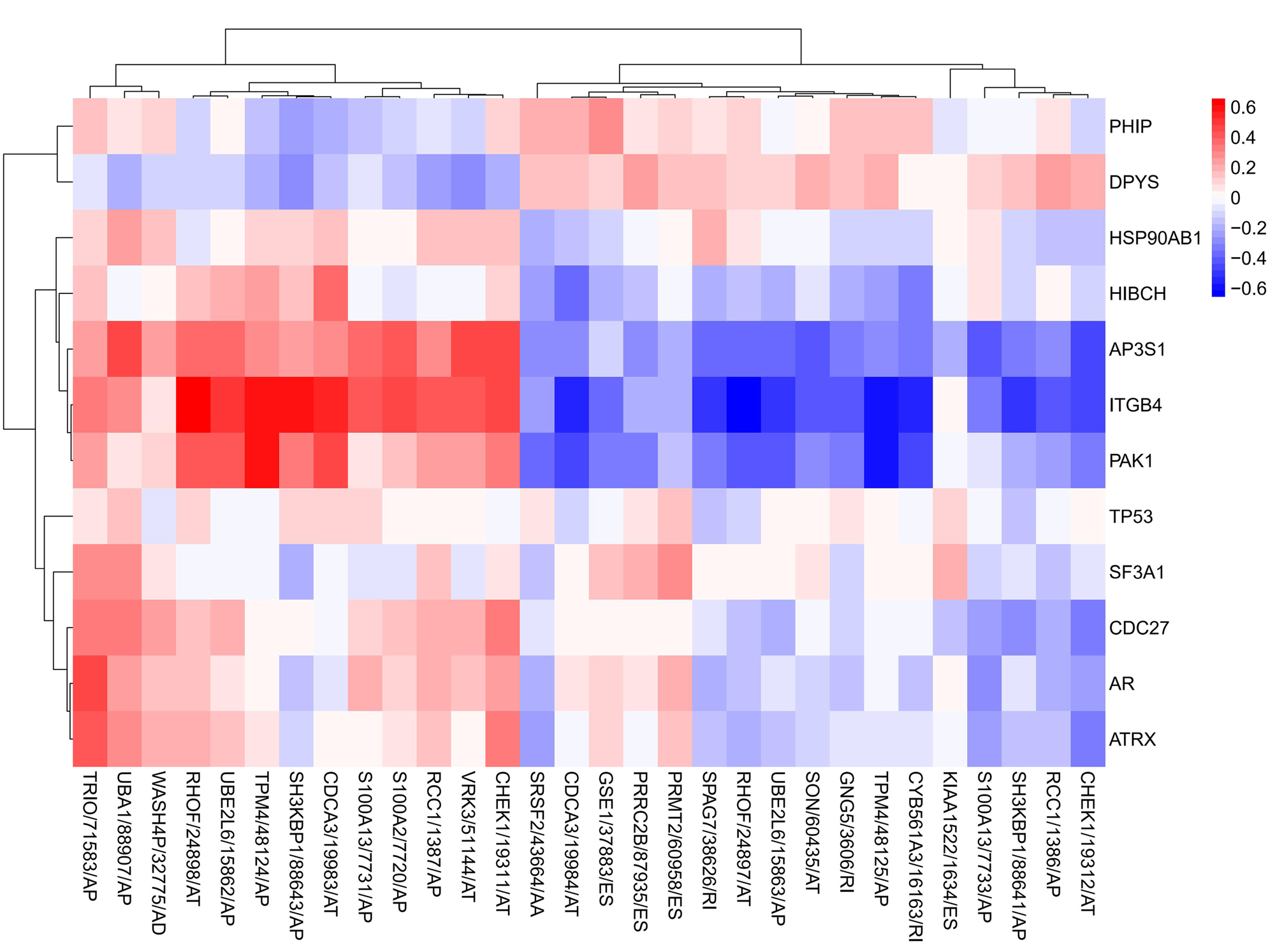

Supplement: Supplemental Figure 3 — The heatmap of the correlations between the mRNA expression of driver genes and PSI values of top 30 survival-associated AS events. Colors represented the correlation coefficient r. [file Image_3.TIF]

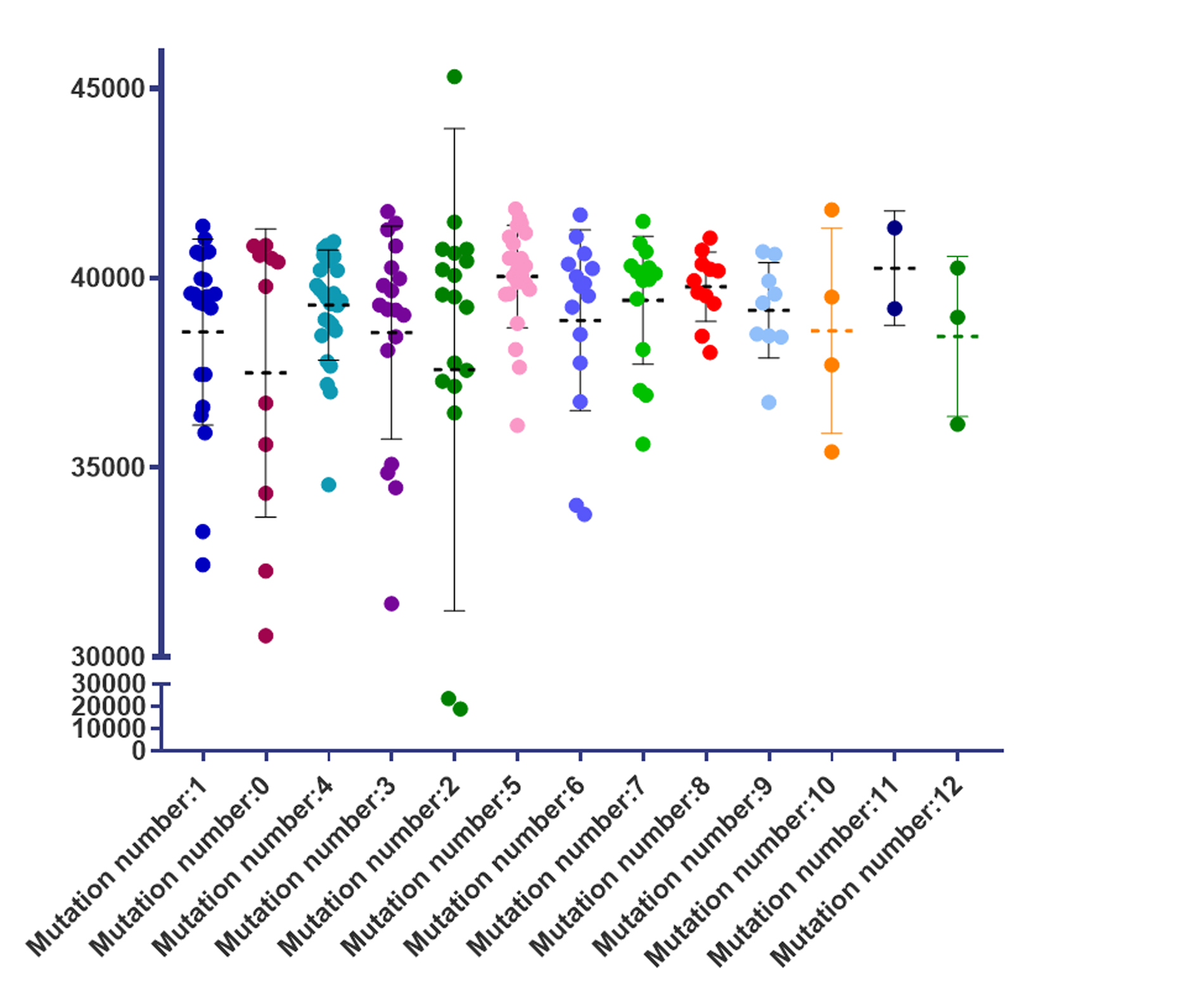

Supplement: Supplemental Figure 4 — Samples from PAAD cohort were divided into several groups according to numbers of driver gene mutations from 0 to 12 in X-axis. No sample has thirteen gene mutations concurrently. The Y-axis represents the numbers of AS events of each sample. [file Image_4.TIF]
