# Supplementary material for: Genome-Wide Profiling of Prognostic Alternative Splicing Pattern in Pancreatic Cancer
Source: Front Oncol. 2019 Aug 27;9:773. doi: 10.3389/fonc.2019.00773 (PMC6736558; doi:10.3389/fonc.2019.00773)
Supplement: Supplementary Table 1 — Baseline characteristics according to TCGA Clinical data. [file Table_1.DOCX]

**Supplementary Table 1. Baseline Characteristics according to TCGA Clinical data (n=178)**

| **Characteristics** | **No. of Patient (%)** |
| --- | --- |
| Age (y) |  |
| <60 | 59(66.9) |
| ≥60 | 119(33.1) |
| **Gender** |  |
| Male | 98(55.1) |
| Female | 80(44.9) |
| **Vital statue (at follow up)** | |
| Death | 93(52.2) |
| Alive | 85(47.8) |
| **T classification** |  |
| T1 | 7(3.9) |
| T2 | 24(13.5) |
| T3 | 142(79.8) |
| T4 | 3(1.7) |
| Tx | 2(1.1) |
| **N classification** |  |
| N0 | 49(27.5) |
| N1 | 120(67.4) |
| N1b | 4(2.2) |
| Nx | 2(2.2) |
| **Metastasis** |  |
| M0 | 80(44.9) |
| M1 | 4(2.2) |
| Mx | 94(52.8) |
| **Stage classification** |  |
| I A | 6(3.4) |
| I B | 15(8.4) |
| II A | 28(15.7) |
| II B | 119(66.9） |
| III | 3(1.7) |
| IV | 4(2.2) |
| **chronic pancreatitis (history)** | |
| Yes | 13(7.3) |
| No | 129(72.5) |
| Not Available | 12(6.7) |
| Unknow | 24(13.5) |
| **diabetes (history)** |  |
| Yes | 38(21.3) |
| No | 109(61.2) |
| Not Available | 7(3.9) |
| Unknow | 24(13.5) |
